# From research to practice: bridging the implementation gap on the use of tranexamic acid in total knee arthroplasty

**DOI:** 10.1186/s13018-025-05475-y

**Published:** 2025-01-30

**Authors:** Robin M. Pfister, Benjamin F. Pfister, Ronald L. Hager, Nathan Sandholtz, Daniel Abulafia, David Bradshaw

**Affiliations:** 1 Hunter New England Health District, New Lambton Heights, NSW 2305 Australia; 2 Central Coast Local Health District, Gosford, NSW 2295 Australia; 3 Department of Exercise Sciences, College of Life Sciences, Brigham Young University, Provo, UT 84602 USA; 4 Department of Statistics, College of Computational, Mathematical, and Physical Sciences, Brigham Young University, Provo, UT 84602 USA

**Keywords:** Implementation lag, Clinical uptake, Tranexamic acid, Total knee arthroplasty, Evidence based medicine

## Abstract

**Background:**

The use of intravenous tranexamic acid (TXA), an antifibrinolytic agent, has been shown to effectively reduce total blood loss and transfusion rates in total knee arthroplasty (TKA). The aim of this paper is to evaluate the implementation lag and clinical uptake of the use of TXA for primary TKA after publication of two landmark studies. Additionally, it assessed the efficacy of TXA use in TKA in reducing post-operative blood transfusions and hospital length of stay (LOS).

**Methods:**

A total of 763 patients aged over 18 years of age underwent primary TKA at a level 4 metropolitan hospital in Australia between January 2011 and December 2017. Primary outcome measure was use of TXA at operative induction. Secondary outcome measures were post-operative blood transfusion, haemoglobin levels and in-hospital length of stay.

**Results:**

The rate of TXA uptake was ≥ 50% by April–June 2013, 1.5 years following landmark paper publication. TXA use was ≥ 90% by April–June 2015, equating to 3.5 years after landmark publication. For each additional year since publication, the odds that TXA was used in a TKA surgery increased by 254.3%, 95% CI (confidence interval) [195.2%, 334.1%]. There was a negative association between TXA use and blood transfusion rate (p < 0.001), while controlling for other variables. TXA use reduced the odds of blood transfusions occurring by 73.5%, 95% CI [35.8% and 89.8%]. Analysis showed that reduced LOS was seen even after controlling for post-operative blood transfusion (p < 0.05).

**Conclusion:**

The implementation lag from research to clinical practice, using ≥ 90% TXA use in TKA as a proxy, was 3.5 years. The use of TXA reduced LOS and blood transfusion rate in TKA patients.

**Supplementary Information:**

The online version contains supplementary material available at 10.1186/s13018-025-05475-y.

## Introduction

Primary total knee arthroplasty (TKA) is a treatment option for the management of severe knee osteoarthritis [1]. With an increasing rate of obesity and an ageing population, the prevalence of TKA is rising in Australia and worldwide [2]. Peri-operative bleeding is one of the main concerns of TKA due to the risks and costs associated with blood transfusions and increased hospital length of stay (LOS) [3]. The use of intravenous tranexamic acid (TXA), an antifibrinolytic agent, has been shown to effectively reduce total blood loss and transfusion rates in TKA [3].

Two meta-analyses published in 2011 and 2012 were landmark papers substantiating the positive impact of tranexamic acid in primary TKA. Alshryda et al. published their findings in the British Journal of Bone and Joint Surgery in 2011, with a meta-analysis of eighteen trials revealing that intravenous TXA significantly reduced patients requiring blood transfusions as well as reducing peri-operative blood loss [4]. Similarly, Yang et al. published a meta-analysis of fifteen trials in the American Journal of Bone and Joint Surgery in 2012, reinforcing that use of TXA reduces blood loss and blood transfusion rates in TKA [5]. Importantly, the evidence from both studies did not support an increased risk of deep vein thrombosis or pulmonary embolism [4, 5].

Today the use of intravenous TXA in primary TKA is common practice among clinicians and recommended in clinical practice guidelines [6]. Despite this published evidence, there is no indication of the time it took for intravenous TXA to become routinely used in primary TKA. The time taken for published evidence to be widely incorporated into clinical practice is referred to as ‘knowledge translation’ [7, 8]. The factors that affect this process are varied and complex, including time and journal of publication, methods of distribution and the culture characteristics of the target audience [7]. Balas and Boren performed an analysis of the time taken to uptake of evidence-based medicine in procedures and treatments that are currently considered standard care. They estimated an average of 9.3 years as the transition period needed to implement evidence from reviews and textbooks for nine common clinical procedures [9, 10]. However, with landmark original clinical trials they calculated an astounding 15.6 years to reach 50% clinical practice from zero use at time of publication [9]. Similarly, a study investigating the implementation lag of evidence-based therapies in acute coronary syndrome reported a 14-year transition period from guideline recommendation to 90% practice uptake [11].

The current study is the first Australian paper to assess the implementation lag of evidence-based medicine in orthopaedic practice. We have used the uptake of intravenous tranexamic acid use for primary total knee arthroplasty in a Level 4 metropolitan peripheral hospital as a proxy to assess the implementation lag of pivotal clinical research supported in a landmark paper into routine orthopaedic practice in a peripheral Australian hospital.

## Methods

Two papers, deemed landmark studies for TXA use in primary TKA based on journal impact factor > 2, high citation rate > 100 and being meta-analytic reviews of randomised controlled trials, were selected for this research. Additionally, these papers influenced practice guidelines published by the *Veterans Health Administration* and *American Academy of Orthopaedic Surgery*. Alshryda et al. was published in the British Journal of Bone and Joint Surgery in December 2011, a well-known orthopaedic journal with an impact factor of 2.8 at year of publication [12]. Yang et al. was published in the American Journal of Bone and Joint Surgery in July 2012 with an impact factor of 3.3 at time of publication [12]. Both papers have had more than 450 citations in literature. These meta-analyses were a convincing summary of previous published research, supporting the use of TXA in primary TKA. The decision was made to commence data collection a year prior to publication of these papers to investigate any potential use of TXA prior to their dissemination.

A total of 763 patients were included in this study, each of whom underwent primary TKA at Wyong Hospital between January 2011 and December 2017. Wyong Hospital is a 284 bed, level 4, metropolitan hospital. Inclusion criteria for all study participants were age ≥ 18 years and primary TKA.

Exclusion criteria were revision TKA, additional procedures performed under the same anaesthetic, and incomplete documentation which would have resulted in incomplete data. Each patient’s hospital records were retrospectively analysed to gather information on demographics, operation date, surgeon, use of TXA intra- or post-operatively, blood transfusion and length of stay. Pre-operative haemoglobin was measured in routine pre-admission clinics and post-operative haemoglobin was measured day one post-operatively.

The primary outcome of this study was to assess the trends in uptake of TXA in this population over the study period. Secondary outcomes were to analyse the relationship between TXA, blood transfusions and LOS.

The statistical software ‘R’ (version 4.3.2) [13] was used to conduct all analyses. Logistic regression was used to evaluate the association between TXA use and time since the publication of the selected landmark papers, accounting for patient demographics and operation characteristics. Logistic regression was also used to assess the relationship between TXA use and blood transfusions and a Cox proportional hazards regression was used to assess the relationship between TXA use and LOS. Features of autologous blood transfusion devices (e.g. presence of reinfusion drains) are not controlled for in the statistical analysis due to inconsistencies in handwritten documentation for the surgeries in our analysis.

## Results

### Demographics

Of the 763 patients, the average age was 69.3 years (SD 9.12) and there was a female predominance of 61.9%. More patients had their right knee replaced (49.8%) than their left knee (38.9%), and 11.3% underwent bilateral TKA. A total of 11 consultant surgeons performed the operations, with one surgeon performing 39.3% of all TKAs.

### Uptake

**Fig. 1:**
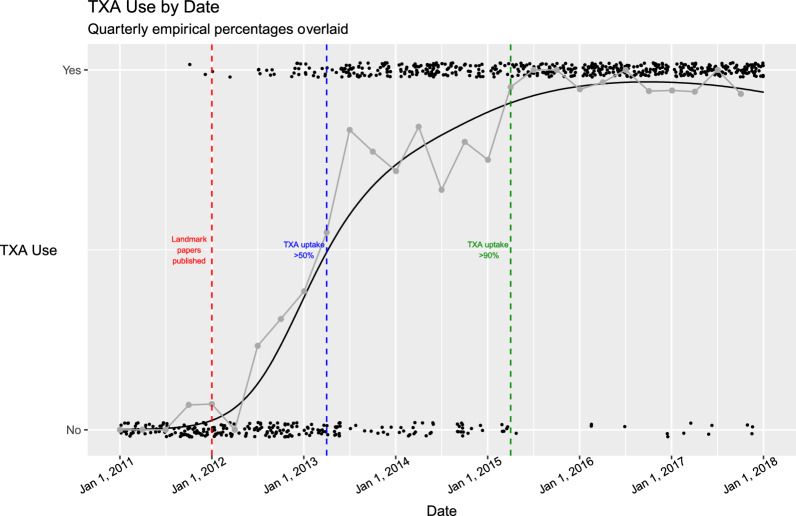
Jittered scatterplot of TXA use (yes/no) by surgery date for all participants in the study. Gray triangles denote the empirical TXA use rates at quarterly intervals. The red line shows the marginal trend of time on TXA uptake (holding other variables constant at their mean values). The shaded region depicts a 95% confidence interval on this trend. Vertical dashed lines mark the date when the landmark TXA papers were published (blue), the date at which the empirical uptake rate exceeded 50% (green), and the date at which the empirical uptake exceeded 90% (orange)

The first of the two landmark papers was published in December 2011. Prior to this there were only two cases in which TXA had been used. Following publication of this study, there was a rapid increase in the use of TXA. The proportions of TXA use at 3-month intervals over the span of our data show a consistent increase in TXA use (Fig. 1, Supplementary Table 1). The rate of TXA uptake was ≥ 50% by April-June 2013, 1.5 years following landmark paper publication. TXA used was ≥ 90% by April-June 2015, equating to 3.5 years after landmark publication (Fig. 1, Supplementary Table 1).

The logistic regression confirmed these apparent trends, showing significant association between time since publication and use of TXA (p < 0.001), accounting for age, gender, bilateral operation (yes/no) and surgeon. On average, for each additional year since publication, the odds that TXA was used in a TKA surgery increased by 254.3%, 95% CI [195.2%, 334.1%], holding all other variables constant.

### TXA and blood transfusion rates

**Fig. 2:**
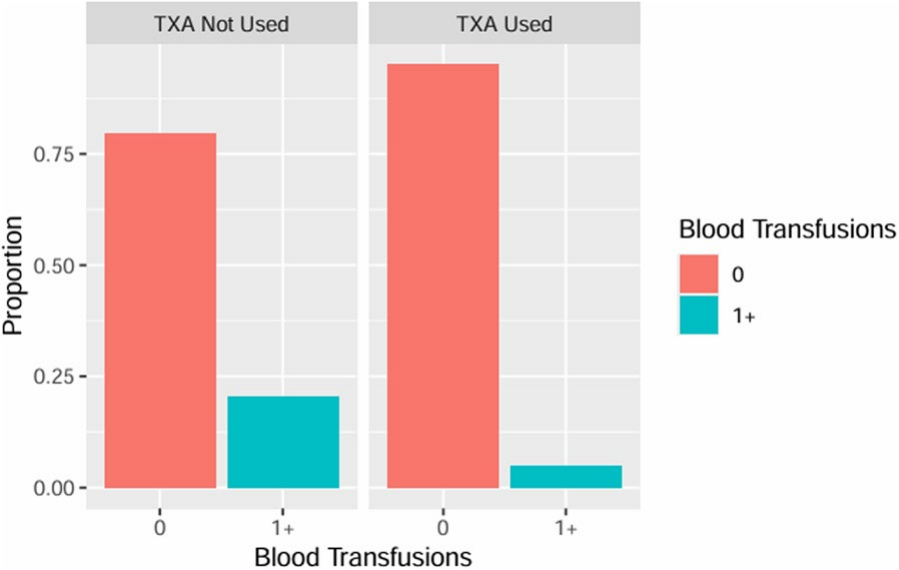
Percentage of TKA surgeries requiring blood transfusions when TXA was not used (left) versus when TXA was used (right)

The logistic regression indicated a significant negative association between TXA use and blood transfusion rate, accounting for time, age, gender, bilateral operation (yes/no) and surgeon (p < 0.001). Specifically, when TXA was used the odds of blood transfusions occurring decreased by 73.5%, 95% CI [35.8%, 89.8%], holding all other variables constant (Fig. 2).

Empirically, the average haemoglobin loss for patients not receiving TXA was 32.89 g/L and the average Hb loss for patients receiving TXA was 27.52 g/L. Linear regression was performed, and results show a clear negative association between TXA use and haemoglobin lost (p < 0.001), accounting for all other variables. Specifically, when TXA is used, the average amount of haemoglobin lost decreases by an estimated 4.59 g/L as compared to TKA surgeries without TXA use, holding all else constant.

### TXA and length of hospital stay

There was a decreased LOS over time with average LOS being 8.4 days prior to publication of the landmark papers, to an average of 5.2 days during the final year of the study. Patients who received TXA had a mean LOS of 5.80 days (SD 6.13) compared to 7.94 days (SD 5.68) in those who did not. To test if there was a statistically significant decrease in LOS for patients who received TXA, we performed a Cox proportional hazards regression on LOS controlling for time, age, gender, surgeon, bilateral operation (yes/no), and blood transfusion (yes/no), finding that TXA indeed reduced hospital length of stay (p < 0.05).

## Discussion

This paper revealed a rapid uptake in the use of tranexamic acid in primary TKA into routine clinical orthopaedic practice. Previous research into the time lag to clinical implementation of evidence-based medicine is limited. In comparison to the reported average 15.6 years by Balas and Boren, this study suggests 18 months to reach > 50% clinical uptake of TXA from time of landmark paper publication [9]. A more recent study purported a 16-year time lag from research publication to > 90% clinical use of medical therapies in acute coronary syndrome [10]. However, our paper revealed a far shorter 3.5 years to achieve > 90% TXA use in TKA from time of landmark paper publication.

The time of publication of these papers must be considered. The former was published in 2000 and investigated the uptake of clinical practices described in literature dating back to 1966. It must therefore be considered that in that time the dissemination of new research took longer than in the current day, and this may have influenced their results. However, the latter paper [11] was published more recently in 2015 and investigated papers and practices between 1990 to 2012. Over those years, the distribution of research and information is more comparable to today’s standards as electronic means of circulation were more readily available.

While it may be that the modern surgical community more effectively circulates new research or that members themselves are more active in remaining up to date and instigating new findings, this is difficult to prove definitively. Rather, as this research was collected at a non-specialised and regional orthopaedic centre it would be interesting to compare these results with a similar study at a specialised, urban centre. Improving the time lag from research to clinical practice will serve to improve patient outcomes with more efficient and higher quality care.

This paper reinforces the knowledge that use of TXA in primary TKA reduces the rate of post-operative blood transfusion. Allogenic blood transfusion following TKA is associated with complications inherent to transfusion as well as poorer post-operative outcomes. In comparison to TKA patients not requiring blood transfusion after surgery, those receiving allogenic transfusion have a higher mortality rate, greater LOS and increased risk of post-operative infection [14, 15]. In addition to this, these patients are more likely to be discharged to an extended care facility and have a greater hospital admission cost [14, 15]. The authors recognise that transfusion practices have evolved in the past two decades with a focus on restrictive transfusion policies supported by a randomised control trial published in 1999 and Cochrane review from 2010 [16, 17]. The authors recognize that the studied hospital did not have a formal transfusion protocol for post-operative TKA during the data collection period. However, as this study took place well after the aforementioned publications regarding restrictive transfusion protocols, we have no evidence to suggest that transfusion practices would confound our analyses.

Our study affirms that the use of TXA reduces in-hospital length of stay. While this could be attributed, in part, to the reduction in post-operative blood transfusions, our analysis showed a reduced LOS even after controlling for post-operative blood transfusion. There are confounding factors that may have influenced the length of stay outcome. For example, in recent years there have been moves to standardise peri-operative TKA care, with particular focus on early mobilisation, which may have had an effect in reducing length of stay. However, the centre at which this study took place did not implement a formal rapid recovery protocol for its knee arthroplasties during the study period. Additionally, the centre did not have a standardized post operative analgesia protocol for TKA patients, nor were these details included in the documentation collected on the surgeries in this study. Whilst the authors have no reason to believe that the post-operative analgesia protocol is correlated with TXA use, this is a factor unaccounted for in the analysis. Analysis indicated a significant negative association between TXA and haemoglobin. Maintaining higher haemoglobin levels may play an important role in improving patients’ post-operative exercise capacity and rehabilitation, thereby ultimately reducing their length of stay [18–20].

The interpretations in this study are limited to TXA use in primary TKA at a single, regional hospital and can therefore only be considered a generic reflection of improvements in the translational process in medical and surgical fields. Also, this study was limited to quantitative analyses and has therefore not investigated the more complex reasons for why time lags to common clinical practice remain.

The authors recognise that a significant proportion of operations were performed by a single surgeon, however our methodology accounted for this potential bias by fitting all regressions as mixed effects models with random effects for surgeon. We also recognise that the practice in TXA use of one surgeon may have influenced the practice of other surgeons at this hospital. While some may argue that this confounds the effect of time to uptake, we feel that it is more logical to consider these effects as the same thing. Uptake time is in many ways a measure of the speed of process of collaboration and interaction that goes on between medical practitioners to ultimately influence their behaviour.

## Conclusion

This study revealed a rapid uptake of intravenous tranexamic acid in primary total knee arthroplasty at a regional, non-specialised orthopaedic centre. This may be considered reflective of the rapidity in which clinicians implement evidence-based medicine in the peri-operative setting after support in a landmark publication. Further research would help solidify this proposition. Studies comparing implementation lags between regional, non-specialised centres and urban, specialised centres would be of interest.

## Supplementary Information

Supplementary file 1.

## Data Availability

Data is stored safely by the authors of this manuscript; it was obtained from Central Coast Local Health District records. The datasets used and/or analysed during the current study are available from the corresponding author on reasonable request. Data analysis and break down of data is provided within the manuscript or supplementary information files.

## Abbreviations

TKA: Total knee arthroplasty
TXA: Tranexamic acid
LOS: Length of stay
