# Supplementary material for: From research to practice: bridging the implementation gap on the use of tranexamic acid in total knee arthroplasty
Source: J Orthop Surg Res. 2025 Jan 30;20:111. doi: 10.1186/s13018-025-05475-y (PMC11780759; doi:10.1186/s13018-025-05475-y)
Supplement: Supplementary file 1 [file 13018_2025_5475_MOESM1_ESM.docx]

Supplementary Table 1: 3 monthly intervals of percentage TXA use.

| Quarter | TXA Rate |
| --- | --- |
| 2011-01 | 0.00 |
| 2011-04 | 0.00 |
| 2011-07 | 0.00 |
| 2011-10 | 0.07 |
| 2012-01 | 0.07 |
| 2012-04 | 0.00 |
| 2012-07 | 0.23 |
| 2012-10 | 0.31 |
| 2013-01 | 0.38 |
| 2013-04 | 0.55 |
| 2013-07 | 0.83 |
| 2013-10 | 0.77 |
| 2014-01 | 0.72 |
| 2014-04 | 0.84 |
| 2014-07 | 0.67 |
| 2014-10 | 0.80 |
| 2015-01 | 0.75 |
| 2015-04 | 0.95 |
| 2015-07 | 1.00 |
| 2015-10 | 1.00 |
| 2016-01 | 0.95 |
| 2016-04 | 0.97 |
| 2016-07 | 1.00 |
| 2016-10 | 0.94 |
| 2017-01 | 0.94 |
| 2017-04 | 0.94 |
| 2017-07 | 1.00 |
| 2017-10 | 0.93 |
